# Clinical Phase I/II trial to Investigate Preoperative Dose-Escalated Intensity-Modulated Radiation Therapy (IMRT) and Intraoperative Radiation Therapy (IORT) in patients with retroperitoneal soft tissue sarcoma: interim analysis

**DOI:** 10.1186/1471-2407-14-617

**Published:** 2014-08-27

**Authors:** Falk Roeder, Alexis Ulrich, Gregor Habl, Matthias Uhl, Ladan Saleh-Ebrahimi, Peter E Huber, Daniela Schulz-Ertner, Anna V Nikoghosyan, Ingo Alldinger, Robert Krempien, Gunhild Mechtersheimer, Frank W Hensley, Juergen Debus, Marc Bischof

**Affiliations:** Clinical Cooperation Unit Radiation Oncology, German Cancer Research Center (DKFZ), Im Neuenheimer Feld 280, 69120 Heidelberg, Germany; Department of Radiation Oncology, University of Heidelberg, Heidelberg, Germany; Department of Surgery, University of Heidelberg, Heidelberg, Germany; Department of Radiation Oncology, Markus Clinic, Frankfurt, Germany; Department of Radiation Oncology, Helios Clinic, Berlin-Buch, Germany; Institute of Pathology, University of Heidelberg, Heidelberg, Germany; Department of Radiation Oncology, SLK Clinic, Heilbronn, Germany; Department of Radiation Oncology, University of Munich (LMU), Munich, Germany

## Abstract

**Background:**

To report an unplanned interim analysis of a prospective, one-armed, single center phase I/II trial (NCT01566123).

**Methods:**

Between 2007 and 2013, 27 patients (pts) with primary/recurrent retroperitoneal sarcomas (size > 5 cm, M0, at least marginally resectable) were enrolled. The protocol attempted neoadjuvant IMRT using an integrated boost with doses of 45–50 Gy to PTV and 50–56 Gy to GTV in 25 fractions, followed by surgery and IOERT (10–12 Gy). Primary endpoint was 5-year-LC, secondary endpoints included PFS, OS, resectability, and acute/late toxicity. The majority of patients showed high grade lesions (FNCLCC G1:18%, G2:52%, G3:30%), predominantly liposarcomas (70%). Median tumor size was 15 cm (6–31).

**Results:**

Median follow-up was 33 months (5–75). Neoadjuvant IMRT was performed as planned (median dose 50 Gy, 26–55) in all except 2 pts (93%). Gross total resection was feasible in all except one patient. Final margin status was R0 in 6 (22%) and R1 in 20 pts (74%). Contiguous-organ resection was needed in all grossly resected patients. IOERT was performed in 23 pts (85%) with a median dose of 12 Gy (10–20 Gy).

We observed 7 local recurrences, transferring into estimated 3- and 5-year-LC rates of 72%. Two were located outside the EBRT area and two were observed after more than 5 years. Locally recurrent situation had a significantly negative impact on local control. Distant failure was found in 8 pts, resulting in 3- and 5-year-DC rates of 63%. Patients with leiomyosarcoma had a significantly increased risk of distant failure. Estimated 3- and 5-year-rates were 40% for PFS and 74% for OS. Severe acute toxicity (grade 3) was present in 4 pts (15%). Severe postoperative complications were found in 9 pts (33%), of whom 2 finally died after multiple re-interventions. Severe late toxicity (grade 3) was scored in 6% of surviving patients after 1 year and none after 2 years.

**Conclusion:**

Combination of neoadjuvant IMRT, surgery and IOERT is feasible with acceptable toxicity and yields good results in terms of LC and OS in patients with high-risk retroperitoneal sarcomas. Long term follow-up seems mandatory given the observation of late recurrences. Accrual of patients will be continued with extended follow-up.

**Trial registration:**

NCT01566123.

## Background

Local control rates in patients with retroperitoneal soft tissue sarcoma (RSTS) remain disappointing even after gross total resection, mainly because wide margins are not achievable in the majority of patients [1]. In contrast to extremity sarcoma, postoperative radiation therapy (RT) has shown limited efficacy due to difficulties in achieving adequate dose and coverage [2]. Although intraoperative radiation therapy (IORT) has been introduced in some centers to overcome the dose limitations and resulted in increased outcome [3], local failure rates are still high even if considerable treatment related toxicity is accepted [2, 3]. Compared to the postoperative approach, preoperative radiation therapy could offer several benefits, including a more precise target volume definition with smaller safety margins, reduced toxicity to adjacent organs at risk because of their displacement through the tumor itself, a possible devitalisation of tumor cells and the avoidance of treatment delays due to postoperative complications [1]. The use of Intensity-modulated radiation therapy (IMRT) further offers improved target coverage with reduced dose to adjacent organs at risk compared to conventional irradiation [4] and the opportunity to reduce overall treatment time using an integrated boost concept with simultaneously increased dose per fraction to the gross tumor volume. Since little data exists about the combination of these approaches, we initiated this prospective, non-randomised, single center trial [5] to investigate the value of dose-escalated preoperative IMRT followed by surgery with an intraoperative electron boost to reduce the local recurrence rate without a markedly increased toxicity. Due to the slow accrual of patients, we performed an unplanned interim analysis to decide, if the trial should be continued or stopped. The results are presented here.

## Methods

### Study design

**Figure 1:**
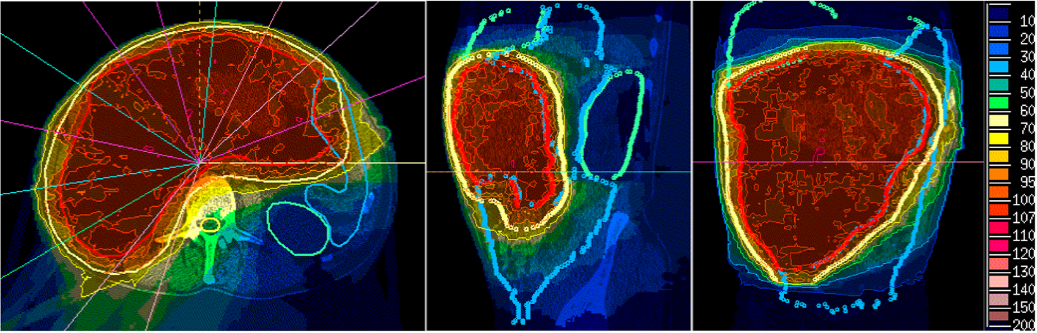
**Example for a dose distribution in a large retroperitoneal sarcoma.** Left: axial view, middle: sagittal view, right: frontal view, red line: GTV, yellow line: PTV, other coloured lines: organs at risk, legend: percentage of prescribed dose.

Details of the study design have been published elsewhere [5]. Briefly, the trial was designed as a prospective single-center one-armed phase I/II study. Inclusion and exclusion criteria are listed in Table 1. Pretreatment evaluation included clinical examination, laboratory tests, histological confirmation, CT or MR-imaging of the abdominal cavity, thoracic CT, bone scan, scintirenography, evaluation of general and technical resectability. For neoadjuvant IMRT, patients were immobilized using an individual body mask system or a vacuum pillow. Inverse treatment planning was based on contrast enhanced CT and MRI. The Gross Tumor Volume (GTV) included all macroscopic tumor. The clinical target volume (CTV) included the GTV with a margin of 1.5 cm in all directions. A safety margin of 5 mm was added to obtain the Planning Target Volume (PTV). Margins could be reduced with respect to anatomical borders or adjacent organs at risk. The attempted dose was 45–50 Gy to the PTV and 50–56 Gy to the GTV in 25 fractions (integrated boost concept). Doses were prescribed to the median of the GTV, while the different target volumes should be surrounded by the corresponding 95% isodose line. An example of a dose distribution in a very large tumor is shown in Figure 1. Treatment was performed using step-and-shoot IMRT after stereotactic target point localisation. Setup correction was done using an image-guided approach with an In-Room-CT on rails at least weekly. Since October 2008 all patients have received daily In-Room-CT pretreatment setup verification and correction. Re-evaluation including restaging with abdominal CT/MRI and assessment of toxicity was scheduled 4 weeks after the last fraction of neoadjuvant irradiation. Gross total resection of the tumor was attempted within 2 weeks from restaging, including contiguous-organ or major vessel resection and reconstruction if necessary. IORT using a dedicated linear accelerator was directed to the whole tumor bed with an attempted dose of 10–12 Gy prescribed to the 90% isodose. If the complete tumor bed could not be covered due to its large size, IORT was directed to the intraoperatively defined high-risk region for positive margins. Definition of the high-risk region was done by the treating surgeon together with the radiation oncologist based on macroscopic evaluation of the resected specimen in correlation with the preoperative imaging. Frozen sections of the tumor bed were generally available but not routinely used. Multiple field IORT was considered and performed only if an overlap of the fields could definitively be avoided. If no coverable high risk region could be defined, IORT was not performed. Regular follow-up visits took place every 3 months after surgery for the first 2 years and every 6 months for three further years. They included at least clinical and laboratory examinations, CT or MR-imaging of the abdominal cavity and thoracic CT (every second visit).

**Table 1:** **Inclusion and exclusion criteria**

Inclusion criteria	Exclusion criteria
• Written informed consent	• Missing written informed consent
• Histologically confirmed, primary or locally recurrent soft tissue sarcoma of the retroperitoneal space	• Missing histological conformation of soft tissue sarcoma
• Judged as at least marginally resectable	• Desmoid Tumors (aggressive fibromatosis), Gastrointestinal Stroma Tumors (GIST)
• Absence of distant metastases	• Judged as gross incomplete or not resectable
• Tumor size ≥ 5 cm	• Incomplete staging
	• Presence of distant metastases
	• Prior radiation therapy to the abdominal region
	• Participation in another clinical interventional study
	• Inflammatory bowel disease

### Statistical and legal considerations

The primary objective was the local control rate after 5 years. Secondary objectives were progression-free survival, overall survival, acute and late toxicity, resectability and patterns of recurrence. Local control was defined as absence of disease progression/recurrence in the abdominal cavity (except organ metastases or diffuse peritoneal sarcomatosis). Progression-free survival (PFS) was determined as absence of local or distant recurrence/progression or death of any cause. Time to event data was calculated from the first day of radiation treatment using the Kaplan-Meier method. Acute and late radiation toxicity was assessed according to Common Terminology Criteria for Adverse Events Version 3.0 (CTCAE 3.0). Postoperative complications were assessed according to the Clavien-Dindo classification [6]. Based on the initial statistical considerations, the calculated sample size was 37 patients in the per protocol population to detect an improvement in the 5-year local control rate from 50% (assumption from the literature after surgery and postoperative 3D-conformal radiation therapy) to 70% with a power of 80%. According to the protocol, data should be analysed separately by per protocol and full set analysis population (defined as all patients who started radiation therapy). The study protocol was approved by the independent ethics committee of the Medical Faculty at the University of Heidelberg. The trial was carried out by adhering to local legal and regulatory requirements. The study complies with the Declaration of Helsinki 2004, the principles of Good clinical practice (GCP) and the German Federal Data Protection Act. Written informed consent was obtained from each patient prior to study entry.

### Patient characteristics

The current interim analysis was based on the full set analysis population after the inclusion of 27 patients between 2007 and 2013. Median age at inclusion was 60 years (range 37–76 years) and 52% of the patients were male. The majority of patients showed high-grade lesions (FNCLCC G1:18%, G2:52%, G3:30%). The predominant histology was liposarcoma (70%). Median tumor size was 15 cm (6–31 cm). For detailed patient characteristics see Table 2.

**Table 2:** **Patient characteristics**

Patient characteristics	n	%
**Age [yrs]**		
Median	60	
Min	37	
Max	76	
**Gender**		
Male	14	52
Female	13	48
**Situation**		
Primary	23	85
Recurrent	4	15
**Histology**		
Liposarcoma	19	70
Leiomyosarcoma	8	30
**Grading (FNCLCC)**		
G1	5	18
G2	14	52
G3	8	30
**Size [cm]**		
Median	15	
Min	6	
Max	31	
**Symptoms at presentation**		
None	4	15
Max grade 1	15	55
Max grade 2	7	26
Max grade 3	1	4

n: number of patients, %: percentage, FNCLCC: Federation Nationales des Centres de Lutte Contre le Cancer, cm: centimeter.

## Results

### Completion of planned treatment

The median follow-up was 33 months (5–75 months) for the entire cohort, and 37 months (8–75 months) in surviving patients. Neoadjuvant IMRT was completed as planned in 25 patients (93%) without treatment breaks > 4 days. In two patients neoadjuvant IMRT was prematurely finished. One suffered from local disease progression and proceeded to immediate surgery after 13 fractions, one developed grade 3 leukopenia and treatment was stopped after 23 fractions to avoid compromise of surgical treatment.

The median GTV and PTV volumes were 1146 ccm (range 62 to 6763 ccm) and 2452 ccm (range 388 to 8516 ccm), respectively. A median number of 9 beams (range 5–14) with 144 segments (43 to 242) was used to deliver median doses of 50 Gy (range 26–55 Gy) to the GTV and 45 Gy (23.4 to 50 Gy) to the PTV (see Table 3).

**Table 3:** **Treatment characteristics**

Treatment	n	%		n	%
**Neoadjuvant IMRT**			**Surgery**		
Completed	25	93	Gross total	26	96
Not completed	2	7	Explo. lap	1	4
**GTV volume [ccm]**			**Resection margin**		
Median	1146		R0	6	22
Min	62		R1	20	74
Max	6763		Explo. Lap.	1	4
**PTV volume [ccm]**			**Cont. organ resection**		
Median	2452		yes	26	96
Min	388		no	1	4
Max	8516				
			Number of organs*		
**GTV dose [Gy]**			0 (explo Lap)	1	4
Median	50		1	7	26
Min	26		2	8	30
Max	55		3	5	19
			4	6	22
**PTV dose [Gy]**					
Median	45		**Type of organ**		
Min	23,4		Nephrectomy	18	67
Max	50		Hemicolectomy	16	59
			Splenectomy	8	30
**Number of beams**			Partial pancreatectomy	8	30
Median	9		Cholecystectomy	8	30
Min	5		Small bowel resection	3	11
Max	14		Rectum resection	3	11
			Partial colpectomy	2	7
**Number of segments**			Partial liver resection	1	4
Median	144		Cystectomy	1	4
Min	43		Adnexectomy	1	4
Max	242		Hysterectomy	1	4
**IOERT**			**Muscle resection**		
Completed	23		Yes	9	33
Not completed	4		No	18	67
			Psoas	4	15
**IOERT dose [Gy]**			Diaphragm	6	22
Median	12				
Min	10		**Vessel resection**		
Max	20		Yes	8	30
			No	19	70
**IOERT energy [MeV]**			Major artery	4	15
Median	8		Major vein	7	26
Min	6				
Max	12		**Nerve resection**		
			Yes	2	7
**IOERT cone size [cm]**			No	25	93
Median	8				
Min	5				
Max	18		*: Cholecystectomy excluded		

n:number of patients, %: percentage, IMRT: intensity-modulated radiation therapy, GTV: gross tumor volume, PTV: planning target volume, ccm : cubic centimetre, Gy: gray, IOERT : intraoperative electron radiation therapy, MeV : mega electron volts, cm : centimetre, explo. Lap. : explorative laparotomy, R0: microscopically negative, R1 : microscopically positive, cont. contiguous.

The median time spans from the end of neoadjuvant radiation treatment to restaging and from restaging to surgery were 28 days (12–38 days) and 11 days (1–33 days), respectively (excluding the patient with immediate surgery due to progression who did not receive formal restaging). The median time from the end of neoadjuvant radiation treatment until surgery was 39 days (range 6–62 days).

Gross total resection was achieved as planned in 26 patients (96%). Final resection margin was R0 in 6 patients (22%) and R1 in 22 patients (74%). One patient was judged non-resectable intraoperatively. Contiguous organ resection to achieve grossly free margins was required in all resected patients (see Table 3). Major vessel resection and reconstruction was needed in 8 patients (30%). Major nerve resections were required in 2 patients and partial resection of the psoas muscle or diaphragm was necessary in 9 patients (see Table 3).

IORT was performed in 23 patients (85%) including the non-resectable patient. One patient received no IORT due to technical reasons and in the remaining three patients a coverable high-risk region could not be defined intraoperatively. The median IORT dose was 12 Gy (range 10–20 Gy) with a median electron energy of 8 MeV (range 6–12 MeV) and a median cone size of 8 cm (range 5–18 cm), see Table 3.

### Oncological endpoints

**Figure 2:**
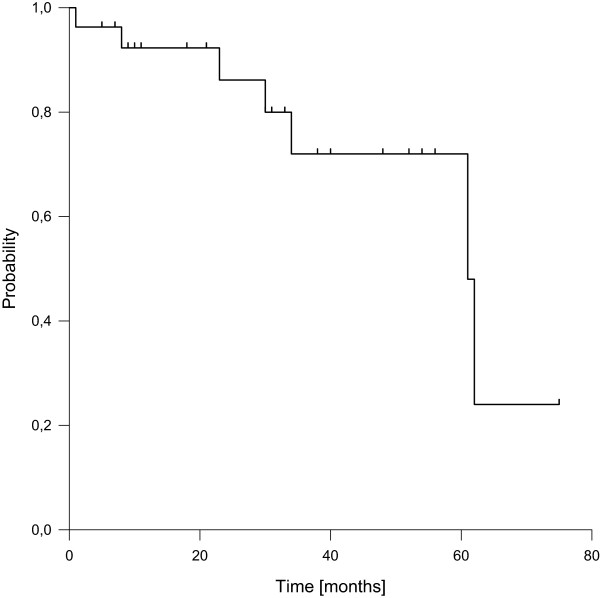
**Local control.**

We found seven local recurrences (26%), transferring into estimated 3- and 5-year local control rates of 72%, respectively (Figure 2). Of note, two of the recurrences were located near but outside the EBRT areas, two were observed after more than 5 years of follow-up and three were found in patients with recurrent situation at inclusion. Recurrent situation at inclusion was the only factor with significantly negative impact on local control in univariate analysis. Patients presenting in primary situation showed estimated 3- and 5-year local control rates of 88%. Local failures were treated by surgery (with or without IOERT) including histological confirmation in 6 patients and with palliative systemic treatment without histological confirmation in one patient with simultaneously diagnosed diffuse metastatic disease. Of the two recurrences observed after more than 5 years, one was histologically confirmed and salvaged by surgery.

**Figure 3:**
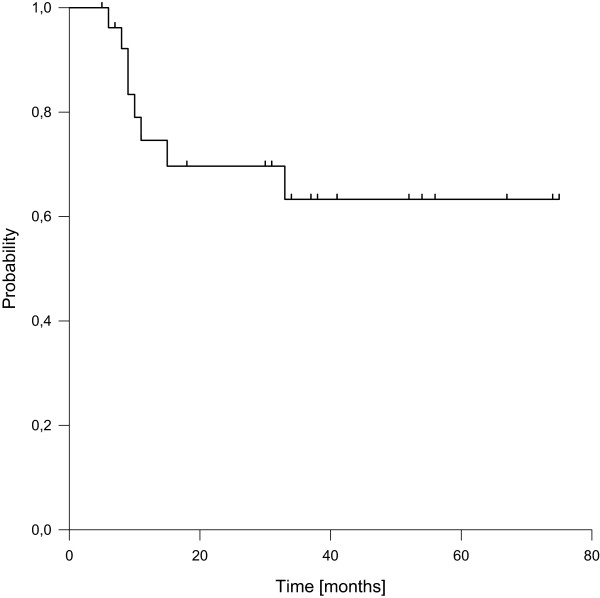
**Distant control.**

Distant failures were observed in eight patients (30%), transferring into estimated 3- and 5-year distant control rates of 63%, respectively (Figure 3). Seven of them occurred during the first two years of follow-up. Histology of leiomyosarcoma was the only factor with significantly negative impact on distant control in univariate analysis.

The 3- and 5-year progression-free survival rates were 40%, respectively. Of the 16 observed events, 5 were isolated local recurrences, 6 were isolated distant failures, 2 were combined failures and 3 were deaths without disease progression. Histology of leiomyosarcoma was the only factor with significantly negative impact on progression-free survival. Furthermore we found a trend for improved PFS in primary vs. recurrent situation.

**Figure 4:**
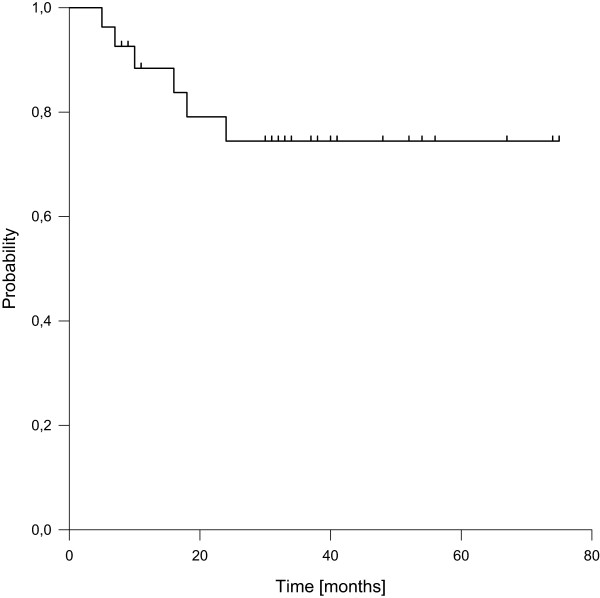
**Overall survival.**

Six patients died during follow-up, transferring into estimated 3- and 5-year overall survival rates of 74% (Figure 4), respectively. None of the tested factors showed a significant impact on overall survival.

### Toxicity

Acute radiation related toxicity was mainly hematological and gastrointestinal. The maximum acute toxicity was grade 1 in 10 patients (37%), grade 2 in 13 (48%) patients and grade 3 in 4 patients (15 %). For detailed analysis see Table 4.

**Table 4:** **Acute toxicity (including the preoperative period)**

Toxicity	All grades	Grade 1	Grade 2	Grade 3
**GI**				
Diarrhea	11	5	4	2
Nausea	11	6	5	0
Appetite loss	4	3	1	0
Bloating	8	3	5	0
Reflux	3	2	1	0
Constipation	3	1	2	0
**Hematological**				
Anemia	18	12	5	1
Leucopenia	9	5	3	1
Thrombopenia	8	8	0	0
**Skin**				
Erythema	10	10	0	0
**GU**				
Frequency/urgency	3	1	2	0
Dysuria	4	3	1	0
Bladder obstruction	1	0	1	0
Ureter obstruction	1	0	0	1
Infection	2	0	1	1
**Other**				
Pain	6	6	0	0
Fatigue	2	1	1	0
Motor neuropathy*	1	0	1	0
Varicocele	1	0	1	0
Hypokalemia	1	1	1	1

GI: gastrointestinal, GU: genitourinary, *:transient.

Severe postoperative complications included bowel/anastomotic leakage, pancreatic fistula/leakage, intra-abdominal bleeding, sepsis and left ventricular dysfunction. The 30-day postoperative mortality rate was 0%, but 2 patients finally died in the prolonged postoperative period. The maximum postoperatively observed toxicity was grade 1 in 7 patients (26%), grade 2 in 10 patients (37%), grade 3a in 5 patients (18%), grade 3b in 1 patient (4%), grade 4b in 1 patient (4%) and grade 5 in 2 patients (7%). 4 patients (15%) needed re-laparotomies (2 of them multiple ones). One of the deceased patients developed a pancreatic/duodenal leakage after partial pancreatic and major vessel resection, but initially denied re-laparotomy and finally died due to massive intra-abdominal bleeding 3 months later although emergency surgery was performed. The other patient developed a pancreatic and multiple bowel leakages with intraabdominal bleeding complications after multi-visceral resection including partial pancreatectomy, nephrectomy, hemicolectomy and splenectomy, and finally died after multiple re-interventions due to septic complications. For detailed analysis see Table 5.

**Table 5:** **Complications in the postoperative period**

Complication	All grades	1	2	3a	3b	4b	5
**GI**							
Functional bowel obstruction	5	1	3	1			
Pancreatic fistula/leakage	5			5**			
Intraabdominal bleeding	3					2	1
Bowel/anastomotic leakage	2					1	1
Bloating/distension	2	1	1				
Constipation	2	2					
Diarrhea	2	2					
Ascites	1			1			
Ulcus ventriculi/duodeni	1		1				
Infection*	2		1			1	
**Wound complication**							
Wound healing disturbance	5	2	1	1	1		
Lymph edema	3	3					
Lymph fistula	1		1				
Decubitus	1	1					
Hematoma	1				1		
**Neuropathy**							
Motor neuropathy/weakness	5	5					
Sensory neuropathy	5	5					
**GU**							
Renal failure	5	5					
Cystitis/infection	4		4				
Ureter obstruction	2	2					
Urinary incontinence	1	1					
Scrotal edema	1	1					
**Cardiac/vascular**							
Av block	1			1			
Ventricular dysfunction	1					1	
Hypertension	1		1				
Deep vein thrombosis	1		1				
**Pulmonary**							
Pleural effusion	2	1	1				
Pneumothorax	1			1			
**Hematological**							
Anemia	2	1	1				
Leucopenia	1	1					

GI: gastrointestinal, GU: genitourinary, *not leakage/fistula associated, **managed with CT-guided drainage procedures.

Observed late toxicity was mainly gastrointestinal, genitourinary and neurological, but generally mild. As late toxicity may vary over time, it was analysed separately at different time points. Patients with salvage treatments were excluded from analysis. Suitable information was available in 16 patients at one year and 14 patients at two years. Maximum toxicity at one year was grade 0 in 4 patients (25%), grade 1 in 7 patients (44%), grade 2 in 4 patients (25%) and grade 3 in one patient (6%). Maximum toxicity at two years was grade 0 in 4 patients (29%), grade 1 in 7 patients (50%) and grade 2 in 5 patients (36%). For detailed analysis see Table 6.

**Table 6:** **Late toxicity**

Toxicity	All grades		Grade 1		Grade 2		Grade 3	
	1 yr	2 yrs	1 yr	2 yrs	1 yr	2 yrs	1 yr	2 yrs
**GI**								
Diarrhea	3	2	3	2	0	0	0	0
Constipation	1	2	1	2	0	0	0	0
Bloating	2	2	0	0	2	2	0	0
**GU**								
Frequency/urgency	1	1	1	1	0	0	0	0
Urinary retention	1	1	1	1	0	0	0	0
Cystitis	1	0	1	0	0	0	0	0
**Other**								
Motor neuropathy/weakness	4	3	1	1	3	2	0	0
Sensory neuropathy	5	3	5	3	0	0	0	0
Lymph edema	4	4	2	3	2	1	0	0
Pain	2	3	2	3	0	0	0	0
Spondylodiscitis	1	0	0	0	0	0	1	0

GI: gastrointestinal, GU.genitourinary, yr: year, yrs: years.

## Discussion and conclusions

The optimal management of retroperitoneal soft tissue sarcomas including the role of radiation therapy has been extensively debated in the past and remains still unclear. In contrast to extremity sarcoma, data from large randomized trials regarding the optimal treatment for retroperitoneal sarcoma is still lacking due to the rarity of this disease and even prospective phase I/II data is rare. Treatment recommendations are mainly based on retrospective single center series, usually covering small patients numbers treated over many years with various combinations of surgical approaches and radiation treatment modalities. Therefore, evidence gained in the much larger studies in extremity sarcoma patients is frequently transferred to guide treatment of retroperitoneal sarcomas. As margin status has been identified as an important prognostic factor in both extremity and retroperitoneal sarcomas [7, 8] surgery with wide negative margins represents the cornerstone of curative intent approaches. However, the achievement of wide or even close negative margins is much more difficult in the retroperitoneal space than in the extremities, resulting in much higher local recurrence rates after surgery alone [9]. Even with more aggressive surgical approaches using en bloc resections of adjacent uninvolved organs, local recurrence remains the dominant pattern of failure in retroperitoneal sarcomas [10, 11]. Additional radiation therapy has clearly been shown to improve local control in extremity sarcomas irrespective of margin status with increasing benefits after close or positive margin resections [7] and is therefore widely accepted as standard of care for these patients. As close or positive margins are more frequent in retroperitoneal sarcomas, this should theoretically lead to an even more pronounced benefit from additional radiation therapy, but only a minority of patients is currently treated with this combination approach [12]. Postoperative external beam radiation therapy was the first RT modality, which has been investigated. But although local control seemed to be improved in many series compared to surgery alone [11, 13, 14], concerns have been raised mainly by the difficulties in achieving adequate dose and target coverage in the postoperative setting. Delivery of efficient doses with generous margins as used in extremity sarcomas is often hampered by the presence of small bowel loops in the resection cavity which would result in unacceptable toxicity in many cases, while reduced doses or margins would compromise efficacy. Because of the known dose-effect relationship which favours doses ≥55 Gy [15, 16], several institutions including ours investigated additional boosting techniques like intraoperative radiation therapy (IORT) or brachytherapy to overcome these limitations [2, 3, 17–19]. The only randomized trial comparing different local treatment approaches in retroperitoneal sarcoma showed a clear benefit favouring a combination of postoperative EBRT (35–40 Gy) and IORT (20 Gy) compared to postoperative EBRT alone (50–55 Gy) in terms of local control (60% vs. 20%) and gastrointestinal toxicity, while neurological toxicity was markedly increased in the IORT arm [3]. Several non-randomized single institution series have confirmed high rates of local control for this combination approach with acceptable neurological toxicities limiting the IORT dose to 15 Gy [2, 17, 18, 20], but there still remains room for improvement regarding both local control and toxicity, especially if compared to extremity sarcomas. Preoperative radiation therapy offers several possible advantages compared to the postoperative approach. These include a possible sterilization of the operative field against seeding, a possible thickening of the often-present pseudocapsule easing resection and the avoidance of repopulation through treatment delays because of postoperative complications. However, the main advantage seems to be the more accurate target volume definition with the possibility of reduced safety margins and reduced toxicity especially to small bowel loops because of their displacement through the tumor itself. Furthermore, the improved oxygenation could increase radiosensitivity and lower the required dose as known from extremity sarcomas. Some of these advantages, especially regarding target coverage and reduction of dose to adjacent organs at risk, can be further exploited with the use of modern radiation techniques like IMRT, VMAT or Tomotherapy as shown in several planning studies [4, 21–23], including the opportunity to reduce overall treatment time by an integrated boost concept.

In our present study, we therefore combined the theoretically advantageous techniques of preoperative intensity-modulated RT with an integrated boost concept, surgery and IORT with the aim to achieve maximal local control. Unfortunately, we faced the same difficulties as many other groups investigating this rare disease, namely poor accrual over a long time span. We therefore decided to perform this unplanned interim analysis to evaluate whether the initial aims of the study are still reasonably achievable. With a median follow-up of 33 months, we found encouraging local control and overall survival rates (estimated 5-year LC 72%, and 5-year OS 74%), especially given the unfavourable patient selection (median tumor volume 1146 ccm and surgery with negative margins possible in only 22% of the patients). Moreover, we found a change in the pattern of failure, with distant metastasis being more prevalent than local recurrences. However, two recurrences were observed shortly after 5 years of follow-up, indicating the need for longer follow up to draw definitive conclusions. Nevertheless, our preliminary results seem to compare favourably with other groups using similar approaches. Pawlik et al. [1] reported a combined analysis of two prospective trials including 72 patients with high grade retroperitoneal sarcomas treated with preoperative radiation therapy and surgery. Preoperative radiation was completed in 89% of the patients and 57 proceeded to surgery. Gross total resection was achieved in 54 patients of whom 32 received an additional boost via IORT or brachytherapy. With a median follow-up of 40 months, they observed 2- and 5-year local control rates of 79% and 60% and a 5-year overall survival rate of 61% after gross total resection. Gronchi et al. [24] reported a prospective trial of 83 patients, who were treated by preoperative radiation therapy combined with chemotherapy. Radiation therapy was completed as planned in 73 and an additional IORT boost was given in 14 patients. 79 patients (95%) underwent surgery. With a median f/u of 58 months, 5-year local control, distant control and overall survival rates were 63%, 74% and 59%, respectively. Smith et al. [25] published the long term analysis of a prospective trial which investigated preoperative radiation in 40 patients combined with selectively applied postoperative brachytherapy. After a median follow-up of 106 months, 5-year overall survival was reported to be 70% with a crude local control rate of 68%. Tzeng et al. [26] reported a small prospective trial investigating dose escalated preoperative IMRT with integrated boost in 16 patients. Gross total resection was achieved in 14 patients, resulting in a 2-year local control rate of 80% after a median f/u of 28 months. These results were also supported by several retrospective series which reported 5-year local control rates of 63-68% and 5-year overall survival rates of 64-72% with similar approaches [27–29], see Table 7. In summary, preoperative radiation therapy followed by surgery with or without additional boost consistently reached high local control and overall survival rates, which seem to be superior to the results of surgery alone or combinations of surgery with postoperative radiation, although formal high level evidence is still lacking. Therefore two phase III trials (ACOSOG Z9031, EORTC 62092) have been designed to evaluate preoperative radiation. While the first trial has already been closed due to poor accrual [27], the results of the ongoing EORTC trial are eagerly awaited and will hopefully clarify the role of preoperative radiation therapy.

**Table 7:** **Series with preoperative radiation therapy**

Author	Year	Type	n	f/u	Pre RT	GTR	Boost	5-year-LC	5-year-OS
Pawlik^1^	2006	Pro.,comb.	72	40	89%	79%	44%	60%*	61%*
Gronchi^24^	2014	Pro.	83	58	88%	95%	17%	63%	59%
Smith^25^	2014	Pro.,subgr.	40	106	100%	100%	48%	63% (cr)	70%
Tzeng^26^	2006	Pro.	16	28	100%	88%	0% (d)	80% (2 yr)	n.s.
McBride^27^	2013	Retro.	33	33	100%	100%	30%	63% (3 yr)	64% (3 yr)
Sweeting^28^	2013	Retro.	18	43	94%	100%	100%	64%	72%
Alford^29^	2012	Retro.	24	28	100%	75%	0%	68%	54%
Present data	2014	Pro.,interim	27	33	93%	96%	85%	72%	74%

pro.: prospective trial, comb., combined analysis, subgr.: subgroup analysis, interim: interims analysis, n: number of patients, f/u: median follow-up in months, pre RT: percentage of patients with completion of preoperative radiation therapy as planned, GTR: percentage of patients in whom gross total resection was achieved, boost: percentage of patients who received an additional boost via IORT or Brachytherapy, d: preoperative radiation therapy was dose escalated, LC: local control, OS: Overall survival, *: in grossly resected patients, cr: crude rate, 2 yr: 2-year rate, 3 yr: 3-year rate.

Besides oncological outcome, every additional therapy comes along with toxicity. Therefore, the possible benefits in terms of local control or overall survival have to be weighed against side effects. In our study, mild gastrointestinal and hematological toxicity was common during preoperative radiation therapy, but only 4 patients (15%) suffered from grade III acute toxicity, which seems quite acceptable given the large radiation fields (median tumor size 15 cm, median PTV ~ 2400 ccm). Jones et al. [30] described GI/pelvic grade I toxicity in 34% and grade II in 47% with no grade III side effects in their prospective trial on preoperative radiation. Pisters et al. [31] reported a dose escalation trial with simultaneously given doxorubicin and described high rates of acute gastrointestinal grade III-IV toxicity (18%) and hematological toxicity (27%) in patients treated at the 50.4 Gy dose level, although at least parts of the side effects might be attributable to chemotherapy. Gronchi et al. [24] also reported higher grade III/IV toxicity rates, but again this trial included simultaneously applied chemotherapy and therefore is difficult to compare. Caudle et al. [32] found acute toxicity in 43% of 14 patients treated with preoperative radiation. From the description, it can be estimated that the rate of severe acute side effects (grade 3 or higher) was 21%, although toxicity was not formally graded. Zlotecki et al. [33] compared patients with preoperative and postoperative radiation and found significantly decreased severe acute side effects after preoperative radiation therapy (36% vs 80%).

Preoperative radiation did not compromise the general ability for surgery in our study, as all patients proceeded to surgery and all except one received gross total resection. However, we observed a considerable rate (33%) of severe postoperative complications, including 2 patients (7%) who finally died after multiple-interventions in the prolonged postoperative period (30 day postoperative mortality rate was 0%). Jones et al. [30] reported severe postoperative complications in 41% of their patients treated with preoperative radiation and selectively applied brachytherapy. They further described a 30 day mortality rate of 2%, but two additional patients (4%) died during the following 18 months due to anastomotic leakage and duodenal perforation. Gronchi et al. [24] observed 21% major postoperative complications after preoperative chemoradiation and Alford et al. [29] found 44% severe postoperative complications after preoperative radiation without additional boosting techniques. These results raise the question, if preoperative radiation increases the postoperative complication rate. However, this question is difficult to answer, as data from prospective series using surgery only is rare. Strauss et al. [34] described a 30 day mortality rate of 3% after surgery alone. Lewis et al. [35] reported a 30 day mortality rate of 4% in a large series of patients treated with surgery only or additional radiation and chemotherapy. Bonvalot et al. reported a mortality rate of 3% with only 18% of their patients requiring an invasive therapeutic procedure and 12% re-operation rate in a series treated with so called “aggressive frontline surgery” [36]. About one third of the patients had received preoperative radiation, but unfortunately the complication rate was not reported separately for the groups with or without radiation treatment. Zlotecki et al. [33] compared pre- and postoperative radiation therapy and observed a significant difference in severe postoperative morbidity (20% vs 53%) favouring the preoperative approach. Finally, Bartlett et al. [37] compared preoperative radiation with surgery alone in a large retrospective series and found no significant differences, neither in overall morbidity and mortality nor in specific side effects. Given the extended surgical approach with contiguous organ resection in our patients, the postoperative complication rate seems acceptable and at least not distinctly increased by preoperative radiation. However, regarding the differences in mortality rates covering different postoperative time spans and the wide range of “severe” postoperative complications reported in the (mainly retrospective) literature after surgery with or without radiation therapy, further clarification is needed by standardized reporting of toxicity data from prospective trials.

Severe late toxicity was uncommon in our study, as only 1 patient (6%) showed grade III late toxicity at one year and none of the patients at two years. Jones et al. [30] reported a 10% severe late toxicity rate at 18 months and Smith et al. [25] described severe late toxicity in 11% of the patients after 18 months in an updated report of the same trial with mature follow-up. Alford et al. [29] observed late toxicities of any grade in 46% of their patients. Interestingly, they described neurological lower limb side effects in 21%, although no additional boost via IORT has been performed, indicating that at least parts of the neuropathy effects usually attributed to IORT treatments might be caused by other reasons. However, the low rate of neuropathy might been explained by the lower IORT doses used in our trial compared to many other series. Based on the early findings reported by Shaw et al., which described a significant association between increased IORT dose and neuropathy rate [20], the IORT dose was limited to 10–12 Gy in our study protocol. Interestingly, Haddock et al. described a significant reduction of neuropathy with intraoperative doses of less than 12.5 Gy in a recently updated series of rectal cancer treated with IORT [38], indicating that our dose constraint seemed reasonable.

Clearly our analysis has some limitations mainly because of its nature of an unplanned interim analysis due to slow accrual. This naturally results in a relatively small number of patients with short follow-up, thus limiting the ability to draw definitive conclusions. Nevertheless, it represents prospectively collected data for the combination of preoperative radiation therapy using IMRT followed by aggressive surgery with contiguous organ resection and intraoperative radiation therapy in this rare disease of retroperitoneal sarcoma and therefore adds valuable information to the small existing body of evidence for this combination approach.

In summary, combination of preoperative IMRT, surgery and IORT resulted in promising 5-year local control and overall survival rates with low rates of acute and late toxicity and acceptable postoperative complications. Long term follow-up seems mandatory given the observation of late recurrences. Accrual of patients will be continued with extended follow-up.
